# Association of Low Serum l-Carnitine Levels with Aortic Stiffness in Patients with Non-Dialysis Chronic Kidney Disease

**DOI:** 10.3390/nu12102918

**Published:** 2020-09-24

**Authors:** Yi-Jen Hsieh, Bang-Gee Hsu, Yu-Hsien Lai, Chih-Hsien Wang, Yu-Li Lin, Chiu-Huang Kuo, Jen-Pi Tsai

**Affiliations:** 1 Division of Nephrology, Hualien Tzu Chi Hospital, Buddhist Tzu Chi Medical Foundation, Hualien 97010, Taiwan; hij@mail.tcu.edu.tw (Y.-J.H.); gee.lily@msa.hinet.net (B.-G.H.); hsienhsien@gmail.com (Y.-H.L.); wangch33@gmail.com (C.-H.W.); nomo8931126@gmail.com (Y.-L.L.); hermit.kuo@gmail.com (C.-H.K.); 2 School of Medicine, Tzu Chi University, Hualien 97004, Taiwan; 3 School of Post-Baccalaureate Chinese Medicine, Tzu Chi University, Hualien 970, Taiwan; 4 Division of Nephrology, Department of Internal Medicine, Dalin Tzu Chi Hospital, Buddhist Tzu Chi Medical Foundation, Chiayi 62247, Taiwan

**Keywords:** aortic stiffness, carotid-femoral pulse wave velocity, chronic kidney disease, l-carnitine

## Abstract

l-carnitine (LC) is a co-factor in fatty acid metabolism; its role with respect to aortic stiffness (AS) associated with chronic kidney disease (CKD) was unclear. Our aim was to investigate associations between serum LC levels and AS in patients with non-dialysis CKD stage 3–5. The AS patients were those with carotid-femoral pulse wave velocities (cfPWV) >10 m/s; those with cfPWV ≤10 m/s were included as controls. Serum LC was measured by liquid chromatography and mass spectrometry. Of 136 CKD patients, the 44 (32.4%) with AS were older, exhibited higher rates of diabetes, and had elevated diastolic and systolic blood pressures (SBP), elevated fasting glucose levels and lower levels of serum LC compared to controls. Multivariable logistic regression revealed that serum LC (odds ratio [OR] = 0.949, 95% confidence interval [CI] 0.911–0.988, *p* = 0.011) and age (OR = 1.055, 95% CI 1.013–1.099, *p* = 0.009) were independent predictors of AS. Multivariable stepwise linear regression revealed significant positive (age and SBP) and negative (serum LC) correlations with cfPWV. The area under the curve of serum LC as a means to predict AS in CKD patients was 0.657 (95% CI 0.571–0.736, *p* = 0.0009). We concluded that low serum LC is a significant predictor of AS in patients diagnosed with CKD.

## 1. Introduction

Cardiovascular disease (CVD) is among the main causes of morbidity and mortality in patients diagnosed with chronic kidney disease (CKD). CVD may be associated with traditional risk factors, including age, hypertension (HTN), and diabetes mellitus (DM); also to be considered are CKD-specific risk factors, including inflammation and oxidative stress [1,2]. l-carnitine (LC), transports long chain fatty acids through the mitochondrial membrane where it promotes β-oxidation as well as the metabolism of acyl and acetyl coenzyme A, thereby providing support for the functioning of the mitochondrial respiratory chain and reducing oxidative stress [3,4]. A recent longitudinal follow-up study of overweight middle-aged individuals revealed changes in pulse wave velocity (PWV) associated with serum levels of l-octanoylcarnitine, which is an intermediate fatty acid and by-product of β-oxidation that serves as a marker for incomplete fatty acid oxidation [5]. Deficiencies of LC had been reported in association with insulin resistance, cardiac complications, functional disabilities, hypotension during dialysis, and erythropoietin-resistant anemia in uremic patients [3,6,7,8].

Aortic stiffness (AS) is the result of aberrant regulation of elastin and collagen, oxidative stress, dysregulated mineral metabolism, and inflammation; AS ultimately leads to impaired perfusion pressure in the coronary arteries which ultimately leads to CVD in patients diagnosed with CKD patients [9,10]. Quantitative evaluation of PWVs is used to diagnose AS; it is currently understood to be a strong predictor of future CVD and mortality in CKD patients via mechanisms that are independent of those associated with traditional cardiovascular (CV) risk factors [9,11]. Likewise, low serum levels of LC have been associated with AS in renal transplant recipients [12]. Recent evidence has revealed that administration of LC supplements may serve to limit these co-morbidities in uremic patients; these findings were associated with diminished levels of markers of inflammation and improvements with respect to nutritional markers that were independent of changes of lipid profiles in patients undergoing hemodialysis (HD) [13,14,15]. Use of LC supplements was also associated with improvements in PWV in this patient cohort [16] and also favorably modulated levels of oxidative stress in patients diagnosed with DM [17] together with lower levels of lipoprotein(a) [18].

Taken together, we hypothesized that LC could have a role in limiting the development of atherosclerosis via its capacity to modulate oxidative stress and inflammation. However, very few published studies have evaluated the relationship between serum levels of LC and AS that develops in patients with CKD. As such, our goal in this study was to evaluate the possibility that serum LC levels may be associated with the development of AS measured by carotid-femoral pulse-wave velocity (cfPWV) in this patient population.

## 2. Materials and Methods

### 2.1. Study Participants

We enrolled 136 participants from patients who were diagnosed with CKD at our medical center in Hualien, Taiwan, from January to December 2016. Based on our review of the medical records, patients with HTN were identified as those with systolic blood pressure (SBP) ≥140 mmHg and/or diastolic blood pressure (DBP) ≥90 mmHg, those who were undergoing treatment with anti-HTN medications; patients with DM were identified as those with a fasting plasma glucose ≥126 mg/dL, and/or those undergoing treatment with oral hypoglycemic medications or insulin. Participants were excluded if they were experiencing an acute infection, acute myocardial infarction, heart failure, and/or malignant disease at the time of blood sampling, or if they were unwilling to provide informed consent. The Research Ethics Committee, Hualien Tzu Chi Hospital, Buddhist Tzu Chi Medical Foundation approved this study (IRB103-136-B). All participants provided their informed consent prior to their participation in this study and were divided into different CKD stages according to the Kidney Disease Outcomes Quality Initiative criteria. Participants were considered to have CKD stage 3, 4, or 5 if estimated glomerular filtration rate (eGFR) = 59–30 mL/min per 1.73 m^2^, 29–15 mL/min per 1.73 m^2^, or <15 mL/min per 1.73 m^2^, individually.

### 2.2. Anthropometric Measurements

All participants were measured by the same operator. Measurements included body weight and height to the nearest half-kilogram or half centimeter, respectively, while wearing light clothing and without shoes. Body mass index (BMI) was calculated as the weight (in kilograms) divided by the square of the height (in meters).

### 2.3. Biochemical Analysis

Fasting blood samples were centrifuged immediately; serum levels of blood urea nitrogen, creatinine, fasting glucose, total cholesterol, triglycerides, calcium, and phosphorus were determined using an auto-analyzer (Siemens Advia 1800, Siemens Healthcare GmbH, Erlangen, Germany). The eGFR was calculated using the CKD-EPI (Chronic Kidney Disease Epidemiology Collaboration) equation.

### 2.4. Determinations of Serum Free l-carnitine Levels by Liquid Chromatography and Mass Spectrometry

Serum levels of free LC were determined by high-performance liquid chromatography and mass spectrometry (LC–MS). To perform this assay, 100 µL of serum and a 50 mM sodium phosphate dibasic heptahydrate solution were mixed using an Eppendorf pipette (1.5 mL). The solution was then extracted by Novum Simplified Liquid Extraction (SLE, Phenomenex, Novum^®^, Torrance, CA, USA) and was eluted in ethyl acetate (1.5 mL). The eluate was evaporated to dryness under nitrogen flow, and the residue was reconstituted in 100 µL of methanol for analysis. The analysis was performed using a Waters e2695 high performance liquid chromatography (HPLC) system that included a single quadrupole mass spectrometer (ACQUITY QDa, Waters Corp, Santa Clara, CA, USA) and equipped with an analytical column (phenomenex Luna^®^C18(2); 5 µm, 250 × 4.60 mm, 100 Å). The temperature of the column was set to 40 °C and the flow to 0.8 mL/min with an injection volume of 30 µL. The LC–MS analyses were performed using a modified method in which the pre-treated samples were evaluated in a positive (i.e., the LC) ion mode electrospray ionization (ESI); the mass spectrometer was then operated in full scan at the speed of 50–450 *m*/*z*. The individual masses of each compound (LC: 162.1 *m*/*z*) were monitored in the single ion recording mode. Data acquisition and processing were performed by the Empower^®^3.0 software. The retention time for free LC was determined at 2.49 min. Quantification of the endogenous compounds was performed by measuring peak areas, followed by comparison to a calibration curve of the standard solutions [12].

### 2.5. Carotid-Femoral PWV Measurements

Carotid-femoral PWV (cfPWV) was measured using applanation tonometry (SphygmoCor system, AtCor Medical, New South Wales, Australia) as previously described [19]. Measurements were performed with participants in a supine position after a minimum of 10-min rest in a quiet and temperature-controlled room. Simultaneous recording with electrocardiogram signals provided an R-timing reference. Pulse wave recordings were performed consecutively at two appropriately spaced superficial arteries (i.e., the carotid-femoral segment). Software was used to integrate pulse wave and electrocardiogram data to calculate the mean time difference between pulse waves and R-waves on a beat-to-beat basis over an average of 10 consecutive cardiac cycles. The cfPWV was calculated using the elapsed time and distance between the recording sites at the two arteries. Quality indices included in the software were set to ensure uniformity of the data. A cfPWV > 10 m/s was defined as AS; participants with a cfPWV ≤ 10 m/s were classified as controls [20].

### 2.6. Statistical Analysis

Continuous variables were examined for normal distribution by the Kolmogorov–Smirnov test. Data are presented as the mean ± standard deviation (SD) or median with interquartile range (IQR) depending on demonstration of normal distribution. Comparisons between AS and control groups were performed by the Student’s independent *t*-test or Mann–Whitney U test (two-tailed). Categorical data were analyzed by the χ^2^ test and are presented as number and percentage. Continuous variables that were not normally distributed were subjected to logarithmic transformation further linear regression analysis. Multivariate linear and logistic regression analyses were used to determine relationships between all variables and cfPWV and to identify independent factors for developing of AS in CKD patients. A receiver operating characteristic (ROC) curve was used to calculate the area under the curve (AUC) in order to identify the best cut-off value of LC to predict AS in CKD patients. A *p*-value < 0.05 was considered as statistically significant. Data were analyzed by SPSS for Windows (version 19.0; SPSS Inc., Chicago, IL, USA).

## 3. Results

The clinical characteristics and medications used by the CKD patients enrolled in our study are presented in Table 1. Of the 136 CKD patients, there were 51 who were also diagnosed with DM (37.5%) and 108 with HTN (79.4%). The 44 (32.4%) patients diagnosed with AS were older (*p* = 0.019); a higher percentage of this cohort was diagnosed with DM (*p* = 0.037) and had higher SBP (*p* = 0.007), higher DBP (*p* = 0.039), and higher levels of fasting glucose (*p* = 0.038), but interestingly, lower levels of serum LC (28.50 μg/mL [IQR 23.16–36.59 μg/mL] vs. 35.11 μg/mL [IQR 26.38–44.27 μg/mL], *p* = 0.003) than those in the control group. Medications prescribed for CKD patients both with and without AS included angiotensin receptor blockers (ARB, *n* = 59; 43.4%), β-blockers (*n* = 33; 24.3%), α-blockers (*n* = 18; 13.2%), calcium channel blockers (CCB, *n* = 54; 39.7%), statins (*n* = 67; 49.3%), and fibrates (*n* = 10; 7.4%). There were no significant differences in gender, diagnosis of HTN, or use of medications between patients with and without AS.

After adjusting for factors including age, DM, SBP, DBP, fasting glucose, and LC, the results of multivariate logistic regression analysis revealed that both serum LC (odds ratio [OR] = 0.936, 95% confidence interval [CI] 0.893–0.980, *p* = 0.005) and aging (OR = 1.053, 95% CI 1.011–1.097, *p* = 0.013) were both independent factors that were significantly associated with the development of AS (Table 2).

Simple linear regression analysis revealed that logarithmically transformed cfPWV correlated positively with age, DM, SBP, DBP (*p* values all <0.05), as well as negatively with eGFR and logarithmically transformed serum levels of LC. Multivariate force entry method linear regression analysis was performed after adjusting for factors including age, DM, SBP, DBP, fasting glucose, total calcium, phosphorus, eGFR, and LC; these findings revealed that aging (β = 0.197, *p* = 0.025), eGFR (β = −0.191, *p* = 0.042), phosphorus (β = −0.216, *p* = 0.013), and logarithmically transformed serum LC levels (β = −0.243, *p* = 0.003) were all significantly correlated with logarithmically transformed cfPWV (Table 3).

**Figure 1:**
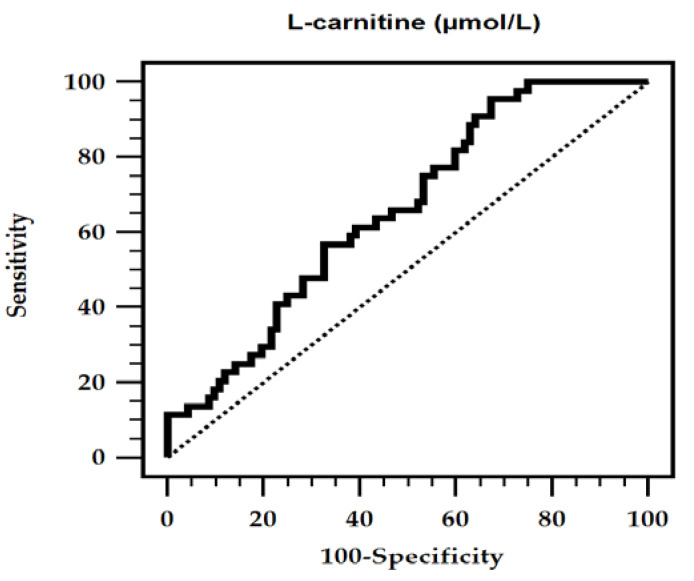
Receiver operating characteristic (ROC) curve analysis of l-carnitine to predict aortic stiffness in chronic kidney disease patients. The area under the ROC curve (AUC) for l-carnitine level was 0.657 (95% confidence interval: 0.571–0.736, *p* = 0.0009).

By using the ROC curve, the best cut-off serum value of LC that can be used to predict AS was 40.21 µmol/L, with an AUC of 0.657 (95% CI 0.571–0.736, *p* = 0.0009), sensitivity of 95.45% (95% CI 84.5–99.4%), and specificity of 32.61% (95% CI 23.2–43.2%) as shown in Figure 1. The clinical variables of the CKD patients divided by low and high l-carnitine value as shown in Supplementary Table S1.

## 4. Discussion

The major findings presented in this study are that both aging and lower levels of serum LC were associated with high cfPWV; as such, these factors may be significant predictors of AS in patients diagnosed with CKD.

A variety of risk factors are known to promote irreversible changes to the vascular wall that result in increased pulse pressure, low impedance circulation, as well a predisposition to developing high blood pressure and mechanical strain; these factors all may contribute to AS and future CVD, renal dysfunction, and mortality [9,10]. Evidence had shown that PWV increases with aging and with elevated BP [21]. Aging might also be associated with anatomical and functional abnormalities of vasculature of patients with CKD; this may result in elastin fragmentation and calcification of the medial layer along with marked age-associated increased in aortic PWV and a decrease in the aortic taper and lower brachial/aortic stiffness gradient [9,22]. The vessel lumen diameter is reduced as AS progresses; this results in the premature return of the reflected wave in late systole and increased pulse pressure, SBP, and decreased DBP [23]. Studies have revealed that cfPWV was positively correlated with high BP and older age in patients who had HTN and DM but had maintained normal renal function [24,25]. Moreover, a systematic review of the literature revealed that age and BP were consistently identified (at 91% and 90%, respectively) as independent risk factors associated cfPWV; interestingly, these studies identified either small or insignificant associations of cfPVW with traditional risk factors such as gender, dyslipidemia, smoking, and BMI [26]. As glucose tolerance deteriorated, an independent association with decreasing arterial compliance, carotid-femoral transit time, and increased aortic augmentation index was revealed; these results demonstrated increased peripheral and central AS in a cross-sectional population study [27]. In the Baltimore Longitudinal Study of Aging, the levels of advanced glycation end products were significantly associated with aortic PWV independent of age, gender, BP, or fasting glucose levels [28]. Moreover, the duration of DM was also independently associated with cfPWV independent of age, gender, BP, or renal function and led to an increasing prevalence of CV events [29]. In a meta-analysis that encompassed 2316 patients, a significant association between DM and aortic PWV was revealed [30]. CKD may be a risk factor for the increased incidence of AS; a recent study reported a eGFR less than 60 mL/min/1.73 m^2^ among patients with CKD was associated with higher brachial-ankle PWVs [31]; likewise, eGFR was negatively associated with PWV in patients with CVD without renal disease and in patients with CKD stage 3 and 4 [32,33]. As renal function decreased, there was a dysregulated complex interplay of mineral metabolism, including calcium, phosphate, and calcium x phosphate product, to induce vasculature to pro-calcify phenotype, which together with hypoalbuminemia could impair arterial elasticity and cause AS [13,14,34]. Similarly, we found that older age, higher BP, and co-morbidities including DM and CKD were associated with higher cfPWV. Furthermore, even after adjusting these cofactors, older age, hypophosphatemia, and lower eGFR were consistently identified as independent factors associated with cfPWV; aging was also identified as a significant and independent predictor for the development of a high degree of AS in CKD patients by multivariate linear and logistic regression analysis.

Inflammation plays a significant role in promoting arterial aging; there is growing evidence that suggests that inflammation may be a factor underlying endothelial dysfunction and AS in patients with CKD and end-stage renal disease (ESRD) [35,36,37]. London et al. [36] reported that age-associated AS was more pronounced among those with increased levels of serum C-reactive protein (CRP). In a longitudinal study, baseline inflammatory markers correlated with AS but could not predict future changes [37]; these findings indicated that inflammatory biomarkers might be associated with AS, but other crucial factors may contributed to its development in patients with CKD. In their study focused on a middle-aged and overweight population, Kim et al. [5] reported a positive correlation between AS and increased levels of free fatty acid oxidation, specifically, levels of l-octanoylcarnitine and decanoylcarnitine, which increased in the setting of LC deficiency [38]. Evidence has revealed important beneficial effects associated with the administration of LC with respect to both inflammatory and nutritional status; this intervention results in significantly lower levels of CRP and interleukin 6 as well as increases in transferrin, albumin, and body mass index in patients undergoing HD [13,14,15,39]. LC supplements can also increase the ratio of reduced/oxidized glutathione and glutathione peroxidase activity along with decreased levels of malondialdehyde and protein carbonylation; taken together, these findings suggest that administration of LC could attenuate oxidative stress and promote an enhanced antioxidant status in HD patients [40]. For example, Signorelli et al. [41] provided HD patients with peripheral arterial disease with supplementation with the LC derivative, propionyl-LC as part of a longitudinal study; among their findings, they reported an improved ankle-brachial index along with significant improvements with respect to endothelial biomarkers as indicated by the progressively reductions in serum endothelin-1. Likewise, Higuchi et al. [16] reported that administration of LC supplements had a beneficial effect with respect to decreased brachial-ankle PWV; these findings highlighted its anti-atherosclerotic effects in HD patients with carnitine deficiency. Finally, Lai et al. [12] recently reported the negative relationship between brachial-ankle PWV and serum LC levels in renal transplantation recipients. In view of these findings and together with our study, the negative association between serum LC levels and cfPWV may be the result of inflammation, oxidative stress, and/or endothelial dysfunction, although a precise and detailed mechanism would require further evaluation.

This study includes several limitations. This study includes a cross-sectional design and was conducted at a single center with only a limited number of CKD patients. Moreover, we did not examine the serum markers of inflammation or oxidative stress. As such, we are unable to determine causal relationships between serum LC levels and AS and the possible mechanisms; a study focused on this parameter would require with more patients and a longitudinal design. Likewise, we did not examine the impact of LC supplements; this specific point would require further study to clarify any of its potential beneficial effects with respect to AS.

## 5. Conclusions

This study demonstrated that increasing age as well as low levels of serum LC may serve as predictors of AS of CKD patients. These findings suggest that LC may play a role in mediating the development of AS. Detailed mechanisms may be revealed in future studies.

## Figures and Tables

**Table 1:** Clinical variables of the 136 chronic kidney disease patients with or without arterial stiffness.

Characteristics	All Participants (*n* = 136)	Control Group (*n* = 92)	Aortic Stiffness Group (*n* = 44)	*p*-Value
Age (years)	67.75 ± 12.73	65.99 ± 12.88	71.73 ± 11.71	0.019 *
Height (cm)	158.24 ± 9.69	158.11 ± 9.53	158.50 ± 10.13	0.827
Body weight (kg)	65.38 ± 14.80	65.25 ± 15.72	65.65 ± 12.83	0.885
BMI (kg/m^2^)	25.94 ± 4.41	25.92 ± 4.80	25.98 ± 3.53	0.942
cfPWV (m/s)	8.85 (7.40–10.80)	7.80 (6.70–8.90)	12.45 (10.83–14.75)	<0.001 *
SBP (mmHg)	149.80 ± 26.47	145.59 ± 23.88	158.61 ± 29.60	0.007 *
DBP (mmHg)	84.57 ± 13.92	82.87 ± 12.65	88.11 ± 15.83	0.039 *
TCH (mg/dL)	159.31 ± 43.66	151.17 ± 46.46	163.77 ± 37.22	0.412
Triglyceride (mg/dL)	119.0 (86.00–161.25)	110.50 (86.00–155.00)	130.00 (94.25–177.00)	0.152
LDL-C (mg/dL)	89.29 ± 36.77	88.03 ± 38.78	91.93 ± 32.42	0.565
Fasting glucose (mg/dL)	99.00 (92.00–138.75)	96.00 (92.00–130.25)	107.00 (94.50–156.50)	0.038 *
HbA1c, (%)	6.2 (5.975–7.5)	6.25 (6.0–7.5)	6.2 (5.8–7.85)	0.859
BUN (mg/dL)	33.00 (24.00–44.00)	32.50 (24.00–47.50)	33.50 (24.50–43.75)	0.980
Creatinine (mg/dL)	1.85 (1.43–2.60)	1.85 (1.40–2.80)	1.85 (1.60–2.38)	0.655
Albumin (mg/dL)	4.00 (3.90–4.30)	4.00 (3.90–4.30)	4.00 (3.83–4.30)	0.759
eGFR (mL/min)	31.39 ± 15.15	32.25 ± 15.89	29.60 ± 13.47	0.412
Total Ca (mg/dL)	9.17 ± 1.88	9.22 ± 1.92	9.06 ± 1.83	0.645
Phosphorus (mg/dL)	3.85 ± 0.81	3.91 ± 0.81	3.73 ± 0.81	0.226
l-carnitine (µmol/L)	33.45 (25.22–39.87)	35.11 (26.38–44.27)	28.50 (23.16–36.59)	0.003 *
Female, *n* (%)	67 (49.3)	45 (48.9)	22 (50.0)	0.906
DM, *n* (%)	51 (37.5)	29 (31.5)	22 (50.0)	0.037 *
HTN, *n* (%)	108 (79.4)	75 (81.5)	33 (75.0)	0.379
GN, *n* (%)	38 (27.9)	29 (31.5)	9 (20.5)	0.178
Smoking, *n* (%)	12 (9.0)	7 (7.7)	5 (11.6)	0.456
ARB use, *n* (%)	59 (43.4)	39 (42.4)	20 (45.5)	0.736
β-blocker use, *n* (%)	33 (24.3)	25 (27.2)	8 (18.2)	0.252
α-blocker use, *n* (%)	18 (13.2)	13 (14.1)	5 (11.4)	0.656
CCB use, *n* (%)	54 (39.7)	36 (39.1)	18 (40.9)	0.843
Statin use, *n* (%)	67 (49.3)	45 (48.9)	22 (50.0)	0.906
Fibrate use, *n* (%)	10 (7.4)	8 (8.7)	2 (4.5)	0.386
CKD stage 3, *n* (%)	68 (50.0)	47 (51.1)	21 (47.7)	0.781
CKD stage 4, *n* (%)	41 (30.1)	26 (28.2)	15 (34.1)	
CKD stage 5, *n* (%)	27 (19.9)	19 (20.7)	8 (18.2)	

Values for continuous variables are given as mean ± standard deviation and tested by Student’s *t*-test; variables not normally distributed are given as median and interquartile range and tested by Mann–Whitney U test; values are presented as number (%) and analysis was done using the chi-square test. BMI, body mass index; cfPWV, carotid-femoral pulse wave velocity; SBP, systolic blood pressure; DBP, diastolic blood pressure; TCH, total cholesterol; LDL-C, low-density lipoprotein cholesterol; HbA1c, glycated hemoglobin; BUN, blood urea nitrogen; eGFR, estimated glomerular filtration rate; Ca, calcium; DM, diabetes mellitus; HTN, hypertension; GN, glomerulonephritis; ARB, angiotensin receptor blocker; CCB, calcium channel blocker; CKD, chronic kidney disease. * *p* < 0.05 was considered statistically significant.

**Table 2:** Multivariate logistic regression analysis of the factors correlated to aortic stiffness among 136 chronic kidney disease patients.

Variables	Odds Ratio	95% Confidence Interval	*p*-Value
l-carnitine, 1 µmol/L	0.936	0.893–0.980	0.005 *
Age, 1 year	1.053	1.011–1.097	0.013 *
Female	1.032	0.415–2.563	0.947
Diabetes mellitus, present	1.755	0.639–4.823	0.276
Systolic blood pressure, 10 mmHg	0.990	0.719–1.365	0.953
Diastolic blood pressure, 10 mmHg	1.591	0.871–2.909	0.131
Fasting glucose, 10 mg/dL	1.029	0.940–1.127	0.533
Total calcium, 1 mg/dL	0.947	0.728–1.232	0.686
Phosphorus, 1 mg/dL	0.554	0.290–1.056	0.073
CKD stage 3	1		
CKD stage 4	1.474	0.534–4.151	0.462
CKD stage 5	0.930	0.238–3.635	0.917

Analysis data was done using the multivariate logistic regression analysis (adopted factors: diabetes mellitus, age, systolic blood pressure, diastolic blood pressure, fasting glucose, l-carnitine, total calcium, phosphorus, and CKD stage). CKD, chronic kidney disease. * *p* < 0.05 was considered statistically significant.

**Table 3:** Correlation between carotid-femoral pulse wave velocity levels and clinical variables among the 136 chronic kidney disease patients.

Variables	Carotid-Femoral Pulse Wave Velocity (m/s)			
	Univariate		Multivariate	
	*r*	*p*-Value	Standardized Beta	*p*-Value
Female	−0.079	0.362	−0.074	0.343
Diabetes mellitus	0.176	0.040 *	0.142	0.068
Hypertension	0.071	0.414	–	–
Glomerulonephritis	−0.060	0.485	–	–
Age (years)	0.253	0.003 *	0.197	0.025 *
Height (cm)	0.034	0.693	–	–
Body weight (kg)	0.215	0.148	–	–
BMI (kg/m^2^)	0.141	0.102	–	–
SBP (mmHg)	0.410	<0.001 *	0.255	0.075
DBP (mmHg)	0.263	0.002 *	0.039	0.786
TCH (mg/dL)	−0.014	0.874	–	–
Log-Triglyceride (mg/dL)	0.112	0.193	–	–
LDL-C (mg/dL)	−0.069	0.424	–	–
Log-Glucose (mg/dL)	0.138	0.109	–	–
Log-Albumin (mg/dL)	−0.041	0.639	–	–
Log-BUN (mg/dL)	0.075	0.383	–	–
Log-Creatinine (mg/dL)	0.147	0.087	–	–
eGFR (mL/min)	−0.197	0.021 *	−0.191	0.042 *
Total calcium (mg/dL)	−0.023	0.795	0.098	0.239
Phosphorus (mg/dL)	−0.073	0.388	−0.216	0.013 *
Log-l-carnitine (µmol/L)	−0.346	<0.001 *	−0.243	0.003 *

Data of carotid-femoral pulse wave velocity, triglyceride, glucose, albumin, BUN, creatinine, and l-carnitine levels showed skewed distribution and therefore were log-transformed before analysis. Analysis of data was done using the univariate linear regression analyses or multivariate forced entry method linear regression analysis (adapted factors were diabetes mellitus, age, SBP, DBP, eGFR, total calcium, phosphorus, and l-carnitine). BMI, body mass index; SBP, systolic blood pressure; DBP, diastolic blood pressure; TCH, total cholesterol; LDL-C, low-density lipoprotein cholesterol; BUN, blood urea nitrogen; eGFR, estimated glomerular filtration rate. * *p* < 0.05 was considered statistically significant.

## Supplementary Materials

The following are available online at https://www.mdpi.com/2072-6643/12/10/2918/s1, Table S1: Clinical variables of the 136 chronic kidney disease patients divided by low and high l-carnitine value.
