# Supplementary material for: Association of Low Serum l-Carnitine Levels with Aortic Stiffness in Patients with Non-Dialysis Chronic Kidney Disease
Source: Nutrients. 2020 Sep 24;12(10):2918. doi: 10.3390/nu12102918 (PMC7598620; doi:10.3390/nu12102918)
Supplement: Supplementary file 1 [file nutrients-12-02918-s001.pdf]

**Supplementary Table.** Clinical variables of the 136 chronic kidney disease patients divided by low and high L-carnitine value

| Characteristics | All Participants (*N* = 136) | Low LC (*N* = 103) | High LC (*N* = 33) | *p*-Value |
|---|---|---|---|---|
| Age (years) | 67.75 ± 12.73 | 68.72 ± 12.92 | 64.73 ± 11.79 | 0.117 |
| Height (cm) | 158.24 ± 9.69 | 156.89 ± 9.74 | 162.42 ± 8.37 | 0.004* |
| Body weight (kg) | 65.38 ± 14.80 | 64.74 ± 15.28 | 67.37 ± 13.21 | 0.378 |
| BMI (kg/m$^2$) | 25.94 ± 4.41 | 26.09 ± 4.45 | 25.46 ± 4.34 | 0.473 |
| cfPWV (m/s) | 8.85 (7.40-10.80) | 9.3 (7.80-11.575) | 7.5 (6.65-8.425) | 0.0002* |
| SBP (mmHg) | 149.80 ± 26.47 | 153.92 ± 27.71 | 136.94 ± 19.89 | 0.001* |
| DBP (mmHg) | 84.57 ± 13.92 | 85.96 ± 14.24 | 80.21 ± 12.05 | 0.039* |
| TCH (mg/dL) | 159.31 ± 43.66 | 158.97 ± 40.98 | 160.36 ± 51.82 | 0.874 |
| Triglyceride (mg/dL) | 119.0 (86.00-161.25) | 119.0 (86.0-165.0) | 116.0 (92.75-146.25) | 0.751 |
| LDL-C (mg/dL) | 89.29 ± 36.77 | 86.56 ± 33.91 | 97.82 ± 44.04 | 0.126 |
| Fasting glucose (mg/dL) | 99.00 (92.00-138.75) | 99.00 (92.0-141.25) | 97.00 (93.0-122.75) | 0.384 |
| HbA1c, (%) | 6.2 (5.975-7.5) | 6.20 (5.9-7.6) | 6.3 (6.0-6.975) | 0.898 |
| BUN (mg/dL) | 33.00 (24.00-44.00) | 32.00 (24.0-44.0) | 38.0 (23.75-60.75) | 0.337 |
| Creatinine (mg/dL) | 1.85 (1.43-2.60) | 1.80 (1.425-2.5) | 2.2 (1.475-3.075) | 0.446 |
| Albumin (mg/dL) | 4.00 (3.90-4.30) | 4.00 (3.90-4.30) | 4.00 (3.90-4.225) | 0.754 |
| eGFR (mL/min) | 31.39 ± 15.15 | 31.11 ± 14.86 | 32.28 ± 16.22 | 0.700 |
| Total Ca (mg/dL) | 9.17 ± 1.88 | 9.23 ± 1.85 | 8.99 ± 2.01 | 0.532 |
| Phosphorus (mg/dL) | 3.85 ± 0.81 | 3.87 ± 0.81 | 3.77 ± 0.84 | 0.535 |
| L-carnitine (μmol/L) | 33.45 (25.22-39.87) | 27.77 (23.91-35.33) | 48.23 (42.37-59.72) | < 0.0001* |
| Female, *n* (%) | 67 (49.3) | 58 (56.3) | 9 (27.3) | 0.007* |
| DM, *n* (%) | 51 (37.5) | 39 (37.9) | 12 (36.4) | 0.959 |
| HTN, *n* (%) | 108 (79.4) | 81 (78.6) | 27 (81.8) | 0.884 |
| GN, *n* (%) | 38 (27.9) | 31 (30.1) | 7 (21.2) | 0.443 |
| Smoking, *n* (%) | 12 (9.0) | 9 (8.7) | 3 (9.1) | 0.749 |
| ARB use, *n* (%) | 59 (43.4) | 41 (39.8) | 18 (54.5) | 0.199 |
| β-blocker use, *n* (%) | 33 (24.3) | 25 (24.3) | 8 (24.2) | 0.818 |
| α-blocker use, *n* (%) | 18 (13.2) | 11 (10.7) | 7 (21.2) | 0.208 |
| CCB use, *n* (%) | 54 (39.7) | 43 (41.7) | 11 (33.3) | 0.512 |
| Statin use, *n* (%) | 67 (49.3) | 54 (52.4) | 13 (39.4) | 0.270 |
| Fibrate use, n (%) | 10 (7.4) | 5 (4.9) | 5 (15.2) | 0.112 |
| CKD stage 3, *n* (%) | 68 (50.0) | 51 (49.5) | 17 (51.5) | 0.914 |
| CKD stage 4, *n* (%) | 41 (30.1) | 32 (31.1) | 9 (27.3) | |
| CKD stage 5, *n* (%) | 27 (19.9) | 20 (19.4) | 7 (21.2) | |

Values for continuous variables are given as mean ± standard deviation and tested by Student's t-test; variables not normally distributed are given as median and interquartile range and tested by Mann–Whitney U test; values are presented as number (%) and analysis was done using the chi-square test.

cfPWV, carotid-femoral pulse wave velocity; DM, diabetes mellitus; HTN, hypertension; BMI, body mass index; TCH, total cholesterol; Ca, calcium; SBP, systolic blood pressure; DBP, diastolic blood pressure; LDL-C, low-density lipoprotein cholesterol; eGFR, estimated glomerular filtration rate; ARB, angiotensin receptor blocker; CCB, calcium channel blocker. *$p < 0.05$ was considered statistically significant.
